# Does rDLPFC activity alter trust? Evidence from a tDCS study

**DOI:** 10.3389/fnins.2023.1213580

**Published:** 2023-09-21

**Authors:** Letian Sun, Xinbo Lu, Haoli Zheng, Lulu Zeng, Wanjun Zheng, Jinjin Wang

**Affiliations:** ^1^Center for Economic Behavior and Decision-Making (CEBD), Zhejiang University of Finance and Economics, Hangzhou, China; ^2^School of Economics, Zhejiang University of Finance and Economics, Hangzhou, China; ^3^School of Economics, Jiaxing University, Jiaxing, China

**Keywords:** trust, dorsolateral prefrontal cortex, trust game, transcranial direct current stimulation, causal relationship

## Abstract

Trust plays an important role in the human economy and people’s social lives. Trust is affected by various factors and is related to many brain regions, such as the dorsolateral prefrontal cortex (DLPFC). However, few studies have focused on the impact of the DLPFC on trust through transcranial direct current stimulation (tDCS), although abundant psychology and neuroscience studies have theoretically discussed the possible link between DLPFC activity and trust. In the present study, we aimed to provide evidence of a causal relationship between the rDLPFC and trust behavior by conducting multiple rounds of the classical trust game and applying tDCS over the rDLPFC. We found that overall, anodal stimulation increased trust compared with cathodal stimulation and sham stimulation, while the results in different stages were not completely the same. Our work indicates a causal relationship between rDLPFC excitability and trust behavior and provides a new direction for future research.

## 1. Introduction

Trust plays an important role in the human economy and people’s social lives. Interpersonal trust is the foundation of deeply understanding social economics, and the lack of trust between trade partners is seriously detrimental to the market economy (Luo, 2018). Trust is a subject of study not only by economists but also by sociologists, psychologists, biologists, and neuroscientists. A large number of behavioral economics and experimental economics studies have shown that in the trust game experiment, both the trustor’s investment and trustee’s transfer are significantly nonzero, which shows that the “economic man” hypothesis has a serious deficiency in explaining trust behavior. This deficiency has led scholars to explore the nature of trust and the factors that influence trust.

There are various factors that affect trust, including preferences (Bohnet et al., 2008; Aimone and Houser, 2011; Aimone et al., 2014), motivations and beliefs (McCabe et al., 2003; Lacour, 2012), oxytocin (Kosfeld et al., 2005; Baumgartner et al., 2008), etc. With the rapid development of neuroimaging and brain stimulation technologies, abundant literature has studied issues, such as trust, cooperation and punishment, with the help of these approaches. Extensive related studies have revealed that brain regions, such as the ventromedial prefrontal cortex (vmPFC), the orbital frontal cortex (OFC), and the dorsolateral prefrontal cortex (DLPFC), have been implicated in trust behavior (Krueger et al., 2007; Sanfey, 2007; Knoch et al., 2009; Phan et al., 2010; Moretto et al., 2013; Zheng et al., 2016; Cheng et al., 2022).

As one of the most important areas in the brain, the DLPFC has received increased attention from some scholars due to its wide range of functions. The DLPFC is an important brain region that is responsible for emotion (Ochsner et al., 2002; Sanfey et al., 2003; Spielberg et al., 2008; Figner et al., 2010; Leyman et al., 2011), and emotion is one of the influencing factors of trust. Engelmann and Fehr (2013) argue that social decision-making (such as trust) also involves emotional considerations that are often overlooked, as there are not only traditional differences but also important links between emotional and cognitive processes. Dunn and Schweitzer (2005) found through behavioral experiments that sad people are less trustful than happy people but more trustful than angry people. Engelmann et al. (2019) reported that aversive affect can have negative impacts on trust behavior in trust games. In trust games, the subjects’ emotions may include betrayal aversion and guilt aversion. Both betrayal aversion and guilt aversion are related to DLPFC activity in trust game (Chang et al., 2011; Aimone et al., 2014).

In addition, neuroscience research has shown that the DLPFC is critical for norm enforcement, especially cooperation and reciprocity (Sanfey et al., 2003; Knoch et al., 2006). Moreover, as an important brain region involved in social cognition, the DLPFC is related to depressive symptoms associated with social cognitive impairment. Some recent fMRI studies have suggested that enhancing DLPFC activity can reduce the severity of symptoms in patients with major depressive disorder (Takamura et al., 2020; Taylor et al., 2022). Fermin et al. (2022) employed neuroimaging techniques and found that low trust was significantly associated with a reduction in DLPFC volume.

There are few studies on the direct relation between the DLPFC and trust behavior. Aimone et al. (2014) implemented a repeated binary investment game and found in a functional magnetic resonance imaging (fMRI) study that compared with computer mediators, participants exhibit more trust when playing a trust game experiment with human peers and found that the rDLPFC, which is related to emotional control, is active. Krueger et al. (2020) argued that in a multiround trust game, the central-executive network (CEN) anchored in the LPFC converts the kindness signal from the default-mode network (DMN) into appropriate reciprocal behaviors fitting for violating norms. Neuroimaging techniques have provided key evidence by revealing changes in the activity of neural networks when humans engage in trust behaviors. This evidence supports the presence of the relationship between neuronal activation and human behaviors but cannot verify the causal relationship in neuroimaging studies. By applying tDCS technology, we can temporarily alter the activity of neurons in related brain areas and verify the causal relationship by observing human behavior changes. Since trust behavior was once viewed as a risk-taking decision linked to DLPFC activity, Zheng et al. (2017) applied tDCS over the rDLPFC and found that enhancing the activity of the rDLPFC alters risk preference without significantly affecting interpersonal trust. However, their experiment was a single-shot trust game. In a repeated trust game, participants’ brain activity becomes more intricate when making decisions due to the effect of their decisions on the entire group’s benefits especially if they pair with different counterparts in different rounds. In this case, the participants must experience role switching and their trust behavior may differ from a single-shot trust game or a repeated trust game that does not include role switching.

In the current study, we applied tDCS (Nasseri et al., 2015; Sellaro et al., 2016) over the rDLPFC to explore whether modulating the excitability of the rDLPFC can directly influence participants’ trust in a trust game. After receiving three different kinds of tDCS stimulations (anodal stimulation, cathodal stimulation or s ham stimulation), participants were required to complete a decision-making task that entailed a multiround trust game. Comparing participants’ trust investment in the trust game under different tDCS stimulations may reveal a causal relationship between rDLPFC excitability and participants’ trust. We also divided the game into several stages to investigate whether there was a different effect of rDLPFC excitability among stages.

## 2. Materials and methods

### 2.1. Subjects

We recruited 120 right-handed and healthy subjects (mean age 21.31 years, ranging from 18 to 27 years; 59 males and 61 females) to participate in our experiment. They were native Chinese-speaking students who declared no history of clinical impairments, neurological disorders, or psychiatric problems. All of the participants were unfamiliar with tDCS and the trust games, had normal or corrected-to-normal vision, and provided written informed consent. All participants, most of whom were undergraduates, came from Zhejiang University of Finance and Economics (ZUFE). Before entering the lab, all participants were required to provide written informed consent approved by the ZUFE Ethics Committee. The experiment was implemented in the Center for Economic Behavior and Decision-making (CEBD) of ZUFE. Each participant received an average payment of 64.02 RMB yuan (approximately 8.77 dollars). No participants reported any adverse side effects related to pain on the scalp or headaches during and after the experiment.

### 2.2. tDCS

The tDCS technology applied a weak direct current via two saline-soaked surface sponge electrodes (5 cm × 7 cm) fixed on the scalps of the participants. Generally, cortical excitability is enhanced when people are receiving anodal stimulation but reduced when people are receiving cathodal stimulation (Nitsche and Paulus, 2000). In our study, we used a tDCS device provided by NeuroConn, Ilmenau, Germany, whose current was stable and not harmful to the participants.

**Figure 1:**
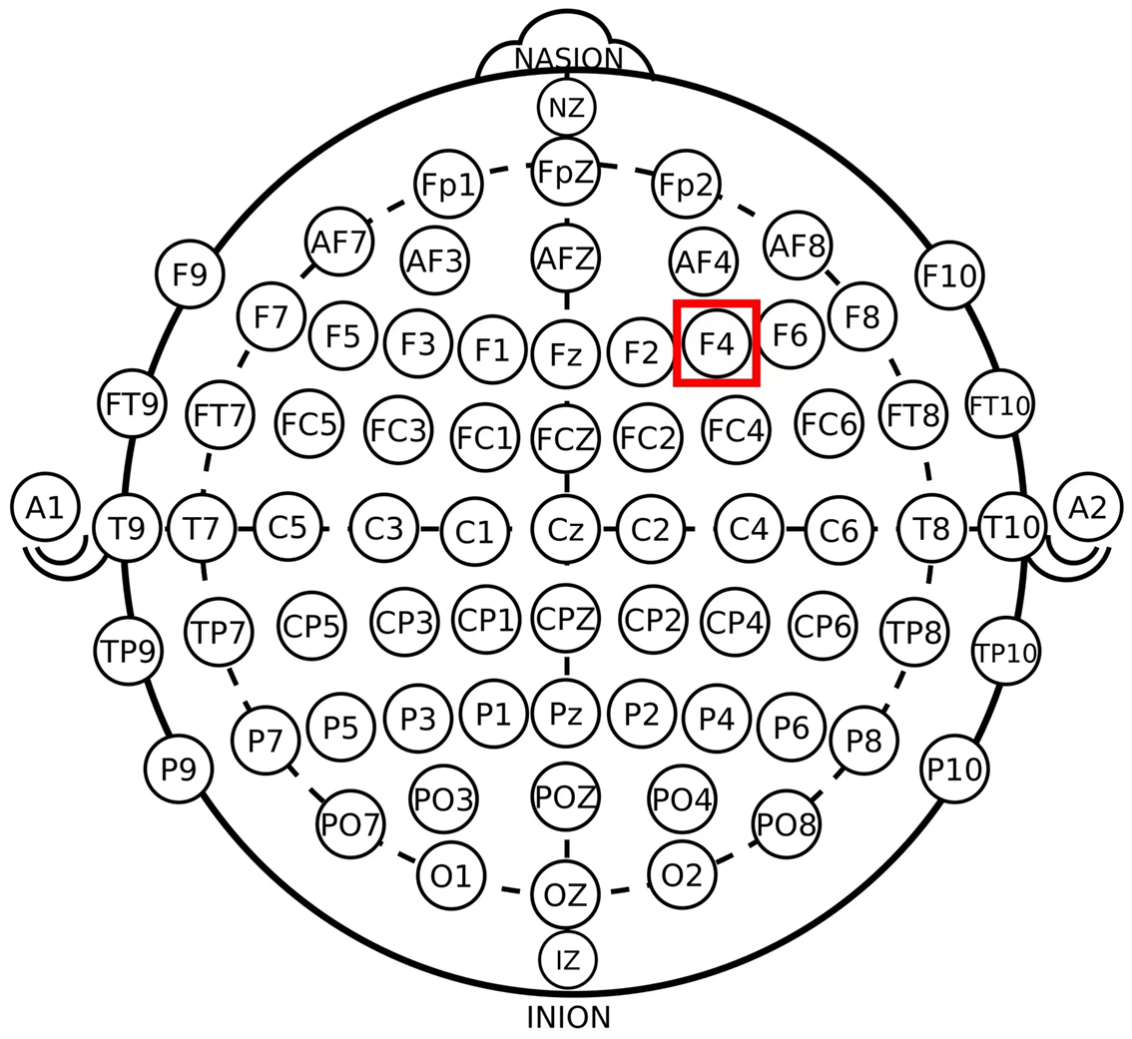
Schematic of the electrode locations of the DLPFC. Schematic of the electrode positions F3 and F4 based on the international EEG 10–20 system.

**Figure 2:**
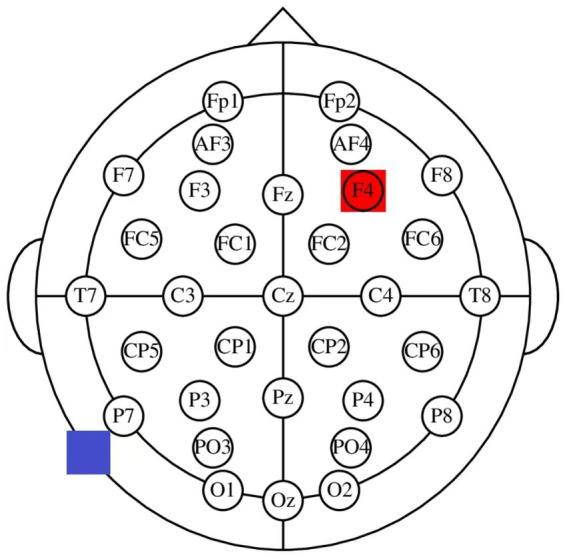
Electrode positions of the rDLPFC.

The participants were randomly assigned to receive one of the three stimulation types: anodal stimulation (*n* = 39, 17 males and 22 females) over the rDLPFC, cathodal stimulation over the rDLPFC (*n* = 40, 22 males and 18 females) and sham stimulation over the rDLPFC (*n* = 41, 20 males and 21 females). According to the international EEG 10/20 system, when receiving anodal stimulation, the F4 position of the participants was the place for the anodal electrode, and the cheek was chosen as the location for the cathodal electrode (Figures 1, 2). For cathodal stimulation, a cathodal electrode was placed over the F4 site, and an anodal electrode was placed over the cheek. For sham stimulation, the stimulation procedure was the same but stopped after 30 s without the subjects’ knowledge, which has been proven to be a reliable approach (Gandiga et al., 2006). The current intensity of stimulation is 2 mA, and the safety and effect of stimulation have been proven in previous studies. Before the experimental task, the laboratory used a strap to fix a sponge sheet on the corresponding target area of the subjects’ heads for stimulation. After 20 min of stimulation, the laboratory members turned off the tDCS device, and subjects were asked to complete a trust game. The game program was executed by z-Tree (Fischbacher, 2007). Given a constant current density, about 10 min tDCS can cause the duration of the excitability changes (Nitsche et al., 2008). And there is research showing anodal tDCS induced effects that last at least 1 h (Goh et al., 2015). Our trust game tasks were all completed within 30 min after tDCS. And considering wearing the tDCS device when playing trust game might have an uncertain impact on the participants’ decision-making, we used offline stimulation.

### 2.3. Experimental design

#### 2.3.1. Trust game

The trust game followed the classical type originally designed by Berg et al. (1995). There are two roles in the trust game: trustor and trustee. The person in each role was offered a certain original endowment (for example, 10 tokens). First, the trustor makes a decision regarding the amount transferred to the trustee. Then, the amount transferred is tripled, and the trustee decides how much of the tripled amount to transfer back to the trustor. For example, if the amount transferred by the trustor is X and the amount returned by the trustee is Y, then the trustor receives (10−X + Y), and the trustee receives (10 + 3X−Y).

In our trust game task, the participants first needed to complete two calculating questions to ensure that they understood the task fully and correctly. The formal task began when all participants accurately answered the question. There were two roles in the trust game, role A (trustor) and role B (trustee). There were 10 rounds in our trust game task. In the 10 rounds, each participant had role A for 5 rounds and role B for 5 rounds. The specific round in which each participant was in role A and role B was pseudorandom. We designed the task so that the participant in role A in round 1 was also in role A in rounds 3, 5, 6 and 9. Therefore, the participant who was in role B in round 1 was also in role B in rounds 3, 5, 6 and 9. At the beginning of each round, the participants were paired randomly by the computer. Each participant was not paired with the same counterpart in different rounds. In each round, participants in role A and role B received 10 tokens as endowment, and the transfer amount of role A shall not exceed 10.

Our trust game experiment has two main differences from other relevant literature. First, our trust game task is a repeated game, which includes 10 rounds. In a single-shot trust game, the participants do not get the opportunity to transfer after experiencing emotion changes. However, in a repeated trust game, before the participants’ transfer decision they may endure negative emotion such as loss, aversion or anger and then make the investment decision. Second, each participant will not be paired with the same counterpart in different rounds and experience role switching. Under these conditions, reputation including learning, updating strategy and building expectations, which may influence trustors’ decision, may not be developed. Both roles assignment and trustor-trustee pairing are random, aiming to isolate the essence of trust and trustworthiness (Alós-Ferrer and Farolfi, 2019).

#### 2.3.2. Procedure

**Figure 3:**
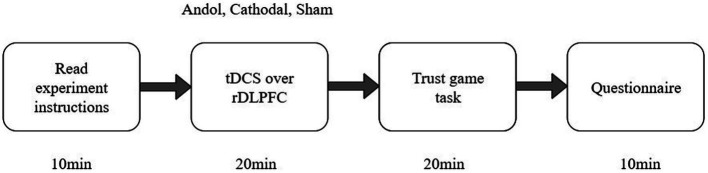
The experimental procedure.

The experimental software z-Tree was used to present the tasks and automatically calculate participants’ final payoffs. The whole experiment contained 3 steps (Figure 3). In step 1, participants were randomly arranged in the seats and received tDCS stimulation for 20 min. In step 2, participants were asked to complete two calculating questions and then played a trust game. In step 3, participants were asked to complete a questionnaire before payment. The questionnaire contained personal information such as sex, age, family income, mother’s education, father’s education, and consumption. In the end, participants received the payment.

### 2.4. Data analysis

#### 2.4.1. Overall analyses

First, we performed a statistical evaluation of the behavioral data using SPSS and STATA software. Specifically, Kruskal-Wallis test was conducted in SPSS software to examine whether there was a significant difference in trust among different stimulation groups over the rDLPFC. The significance level for all analyses was set at 0.05. In our trust game, we defined the amount transferred in the role of the trustor as the participant’s trust. Compared with previous methods of measuring trust through questionnaires, the trust game experiment has become a classic paradigm of trust measurement because of its simple experimental process, effective simulation and simplification of trust in daily life, and does not include any situational variables outside the laboratory. In addition, to evaluate the impact of different stimulations over the rDLPFC on participant trust, we ran regression analysis in STATA software, given by the following equation:


$$y=\beta_{0}+\beta_{1}\times D_{i}+\beta_{j}\times X_{j}+\varepsilon_{i}.$$

The dependent variable is y, which was defined as participant’s trust. Independent variable D_i_ are dummy variables that were set to 1 if individual i received stimulation of anodal, cathodal, or sham, respectively. Thus, the parameter β_1_ quantified the participant’s trust difference between the anodal and cathodal groups or between the anodal and sham groups. X_j_ represents the participant’s personal information, such as sex, age, family income, mother’s education, father’s education, and consumption (Zheng et al., 2021a). To explore the impact of previous payoff as trustee when experiencing role changing, we include what amount trustor get in the previous round as trustee (ltrustee) as a control variable. In addition, we also include what amount trustor transfer on the previous trial (ltrustor) and their cumulative earnings (cumulation) as control variables. The parameter β_j_ captures the effects of control variables under the three stimulation groups.

Table 1 reports the mean (M) and standard error (SE) of the participant’s trust under different stimuli over the rDLPFC.

**Table 1:** Means and standard error of trust under three stimulation conditions.

	M	SE
Anodal	5.98	0.242
Cathodal	4.45	0.261
Sham	4.75	0.267
Total	5.05	0.151

#### 2.4.2. Different stage analyses

Since the trust game was conducted in 10 rounds, it was necessary to compare different rounds to explore whether there were different impacts of stimulation on trust. The specific round in which the participants had role A or role B was pseudorandom, i.e., half of the participants had role A in rounds 1, 3, 5, 6, and 9, and the other half had role A in rounds 2, 4, 7, 8, and 10. Therefore, each participant had role A only once and underwent a role switch in rounds 1 or 2, 3 or 4, 5 or 7, 6 or 8, and 9 or 10 and underwent a role switch. Therefore, we divided the 10 rounds into five stages: rounds 1 and 2 were the first stage, rounds 3 and 4 were the second stage, rounds 5 and 7 were the third stage, rounds 6 and 8 were the fourth stage and rounds 9 and 10 were the fifth stage. In this way, we ensured that each stage would include each participant’s trust data, which was balanced. Kruskal-Wallis tests were conducted to examine each stage separately to study whether the impact of stimulation on the participant’s trust was influenced by the stage. Table 2 reports the M and SE of the participant’s trust in each stage under different stimulations over the rDLPFC.

**Table 2:** Means and standard error of trust in each stage under three stimulation conditions.

	1st M (SE)	2nd M (SE)	3rd M (SE)	4th M (SE)	5th M (SE)
Anodal	5.03 (0.498)	6.21 (0.540)	6.74 (0.524)	6.10 (0.524)	5.82 (0.608)
Cathodal	4.83 (0.576)	4.9 (0.610)	4.75 (0.546)	4.33 (0.583)	3.45 (0.598)
Sham	4.63 (0.512)	5.54 (0.575)	5.29 (0.586)	4.49 (0.672)	3.78 (0.622)
Total	4.83 (0.304)	5.54 (0.333)	5.58 (0.326)	4.96 (0.351)	4.33 (0.362)

## 3. Results

### 3.1. Overall effects of tDCS over the rDLPFC on trust

**Figure 4:**
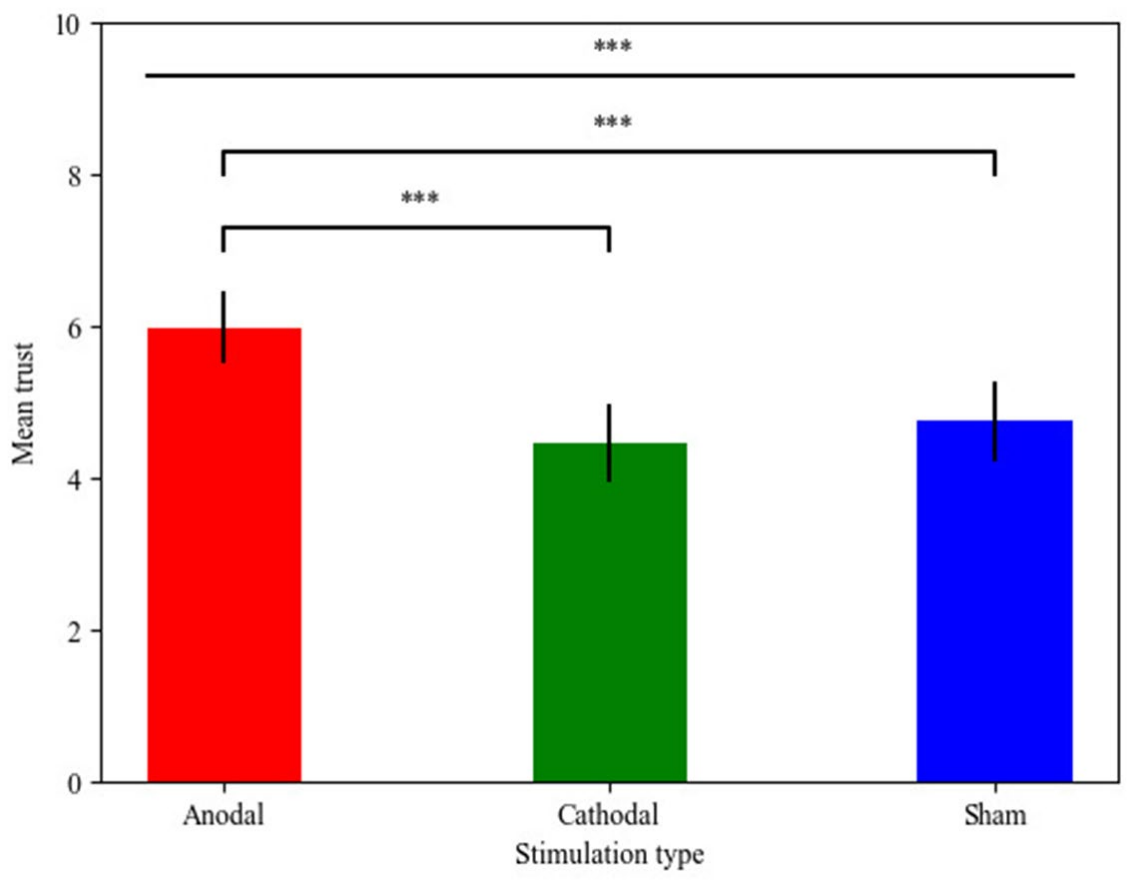
Mean trust in the trust game for the three stimulations of the rDLPFC. The error bars indicate the 95% confidence intervals. The asterisks (^*^) indicate a significant effect at a threshold of *p* < 0.05, the asterisks (^**^) indicate a significant effect at a threshold of *p* < 0.01, the asterisks (^***^) indicate a significant effect at a threshold of *p* < 0.001.

The main goal of our study was to test whether stimulation of the rDLPFC alters trust behavior. For this purpose, the trust data in the trust game under three different stimulation groups (anodal, cathodal and sham) over the rDLPFC was analyzed by means of Kruskal-Wallis test. First, we conducted the Shapiro–Wilk test, a nonparametric method, which revealed that trust in the three different stimulation conditions was not normally distributed (anodal: *p* = 0.760; cathodal: *p* = 0.007; sham: *p* = 0.060). Hence, Kruskal-Wallis test was used to test the stimulation effect. The result revealed that the stimulation over the rDLPFC had significant main effects on participants’ mean trust (χ^2^_d.f.2_ = 19.861, *p* < 0.001, Cohen’s d = 0.351). Specifically, *post hoc* analyses (Bonferroni) revealed that mean trust in the anodal group (mean = 5.98) was significantly higher than that in the cathodal group (mean = 4.45, *p* < 0.001) and sham group (mean = 4.75, *p* < 0.001). However, no significant difference between the cathodal group and the sham group was observed (*p* = 0.473). This result suggests that there is a stimulation effect on the decision to trust and indicates that rDLPFC stimulation works only in the context of anodal stimulation (Figure 4).

To determine the impact of participants’ different characteristics, previous payoffs as trustee, previous trial as trustor and cumulative earnings, an OLS regression model was also conducted through STATA to examine the robustness of the association between rDLPFC and trust behaviors. Table 3 shows the specific stimulation effects in the regression analysis when sham group was set to the base group. For the four regressions, the *F* test that measures the common influence of all independent variables on the dependent variable was all significant (*p* < 0.001; *p* < 0.001; *p* < 0.001; *p* < 0.001). In the first column of the table, we included only participants’ characteristics. We can see that anodal stimulation significantly increased trust compared with sham stimulation, while cathodal stimulation was not significantly different from sham stimulation. In the second column, when controlling previous payoffs as trustee, tDCS effect did not change and previous payoffs as trustee had no significant impact on the next decision as trustor. In the third column, when controlling previous trial as trustor, tDCS effect did not change and previous trial as trustor had a positive impact on the next decision. In the fourth column, when controlling cumulative earnings, tDCS effect also did not change and cumulative earnings had no significant impact on the next decision. Table 4 shows the specific stimulation effects in the regression analysis when cathodal group was set to the base group. The four regressions all show that anodal stimulation significantly increased trust compared with cathodal stimulation. The regression results are consistent with Kruskal-Wallis test, indicating that the subjects who received anodal stimulation were more likely to trust.

**Table 3:** Regression results of tDCS effects on trust (sham group as base group).

Regressor	Base group: sham group			
	1	2	3	4
Anodal	1.208^***^	1.198^**^	0.834^**^	1.216^***^
Cathodal	−0.282	−0.282	−0.185	−0.286
Sham	–	–	–	–
Ltrustee	–	0.010	–	–
Ltrustor	–	–	0.464^***^	–
Cumulation	–	–	–	−0.002
Sex	0.413	0.406	0.194	0.414
Age	−0.179^**^	−0.182^**^	−0.111	−0.179^**^
Medu	−0.268	−0.275	−0.175	−0.264
Fedu	−0.135	−0.136	−0.070	−0.134
Income	−0.072	−0.075	−0.005	−0.068
Consumption	0.054	0.059	−0.012	0.052
Constant	9.386^***^	9.317^***^	5.791^***^	9.473^***^
R-squared	0.056	0.057	0.281	0.056

^***^ *p* < 0.001, ^**^ *p* < 0.01, ^*^ *p* < 0.05.

**Table 4:** Regression results of tDCS effects on trust (cathodal group as base group).

Regressor	Base group: cathodal group			
	1	2	3	4
Anodal	1.491^***^	1.48^***^	1.02^**^	1.501^***^
Cathodal	–	–	–	–
Sham	0.282	0.282	0.185	0.286
Ltrustee	–	0.010	–	–
Ltrustor	–	–	0.464^***^	–
Cumulation	–	–	–	−0.002
Sex	0.413	0.406	0.194	0.414
Age	−0.179^**^	−0.182^**^	−0.111	−0.179^**^
Medu	−0.268	−0.275	−0.175	−0.264
Fedu	−0.135	−0.136	−0.070	−0.134
Income	−0.072	−0.075	−0.005	−0.068
Consumption	0.054	0.059	−0.012	0.052
Constant	9.104^***^	9.034^***^	5.606^***^	9.188^***^
R-squared	0.056	0.057	0.281	0.056

^***^ *p* < 0.001, ^**^ *p* < 0.01, ^*^ *p* < 0.05.

### 3.2. Effects of tDCS over the rDLPFC on trust in different stages

First, the Shapiro–Wilk test showed that in the third stage the trust in the anodal group was not normally distributed (*p* = 0.034). Therefore, Kruskal-Wallis test was performed to determine whether there was a significant difference in trust among different stimulations.

**Figure 5:**
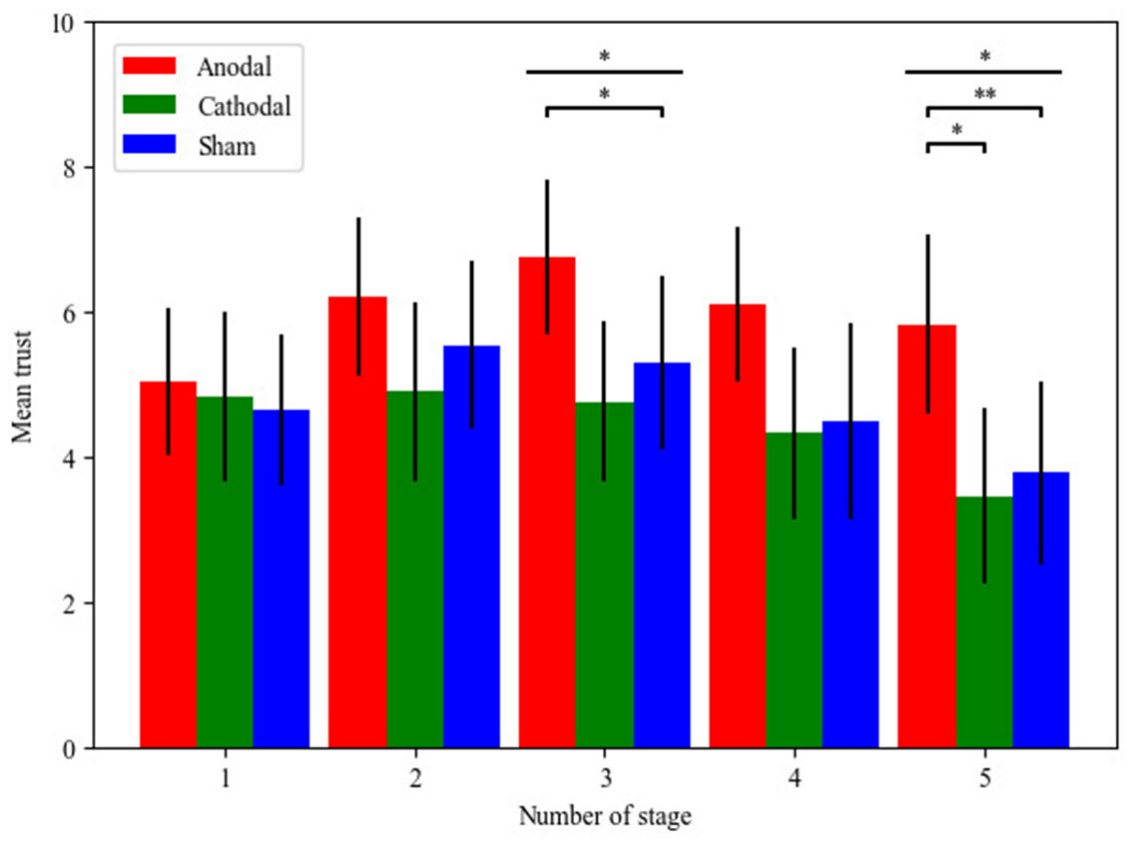
Mean trust of each stage in the trust game across the different stimulations of the rDLPFC. The error bars indicate the 95% confidence intervals. The asterisks (^*^) indicate a significant effect at a threshold of *p* < 0.05, the asterisks (^**^) indicate a significant effect at a threshold of *p* < 0.01, the asterisks (^***^) indicate a significant effect at a threshold of *p* < 0.001.

For the first stage, Kruskal-Wallis test showed that there was no significant main effect of tDCS stimulation (χ^2^_d.f.2_ = 0.565, *p* = 0.754). In the second stage, there was no significant main effect of tDCS stimulation (χ^2^_d.f.2_ = 2.371, *p* = 0.306). In the third stage, there was a significant main effect of tDCS stimulation (χ^2^_d.f.2_ = 6.636, *p* = 0.036, Cohen’s d = 0.406). *Post hoc* analyses (Bonferroni) revealed that trust in the anodal group (mean = 6.74) was significantly higher than that in the cathodal group (mean = 4.75, *p* = 0.012). However, a significant difference was not found between the anodal group and the sham group (mean = 5.29, *p* = 0.074) or between the cathodal group and the sham group (*p* = 0.458). In the fourth stage, there was no significant main effect of tDCS stimulation (χ^2^_d.f.2_ = 5.303, *p* = 0.071). For the last stage, there was a significant main effect of tDCS stimulation (χ^2^_d.f.2_ = 7.979, *p* = 0.019, Cohen’s d = 0.464). *Post hoc* analyses (Bonferroni) revealed that trust in the anodal group (mean = 5.82) was significantly higher than that in the cathodal group (mean = 3.45, *p* = 0.010). There was also a significant difference between the anodal group and the sham group (mean = 3.78, *p* = 0.022). However, no significant difference between the cathodal group and the sham group (*p* = 0.760) was observed. For the first three stages, mean trust in only anodal group increased with stage, while two other groups changed irregularly. And mean trust in the cathodal group and the sham group experienced a sharp decline before the last stage and finally reached the lowest the last stage (Figure 5).

## 4. Discussion

Neuroimaging studies have shown that the DLPFC plays an important role in emotional control and decision-making (Berg et al., 1995; Sanfey et al., 2003; Van Den Bos et al., 2009; Figner et al., 2010; Leyman et al., 2011). Previous neuroscience studies have also revealed a link between the DLPFC and trust behavior (Chang et al., 2011; Aimone et al., 2014; Nihonsugi et al., 2015; Cheng et al., 2022).

In the current study, we applied tDCS over the rDLPFC to determine the impact of rDLPFC excitability on interpersonal trust behavior. We found that activating the rDLPFC could increase trust; that is, the trust of the anodal group was significantly higher than that of the cathodal group and the sham group, providing a causal relationship between the excitability of the rDLPFC and trust behavior.

Although studies have shown that different preferences related to trust have different neural regions, modulating the rDLPFC alone might not be enough to alter trust in humans (Zheng et al., 2017). With the deepening of the research on multiround trust games, the function of the DLPFC, and the relationship between the DLPFC and trust (Alós-Ferrer and Farolfi, 2019; Krueger et al., 2020; Cheng et al., 2022; Fermin et al., 2022), we expected that the application of tDCS over the rDLPFC in multiround trust games might have different results, which was verified in our experimental results.

Our findings indicated that the rDLPFC plays an irreplaceable role in trust behavior. The results showed that activating the rDLPFC could increase trust compared with that in the cathodal group and the sham group. As we discussed in the introduction, previous studies have shown that emotions may affect trust behavior. Dunn and Schweitzer (2005) found through behavioral experiments that people behaved differently according to their emotions. Engelmann and Fehr (2013) believed that negative social interaction in the trust game could induce an aversive affect. These studies did not indicate the neural mechanism involved, but later studies found that the DLPFC is closely linked with disorders concerning emotions (Aimone et al., 2014; Takamura et al., 2020; Fermin et al., 2022; Taylor et al., 2022). Moreover, many studies have shown that DLPFC plays a very important role in controlling negative emotions (Sanfey et al., 2003; Ochsner et al., 2004; Corradi-Dell'Acqua et al., 2013). In our trust game, trustors were inevitably faced with negative results by trustees and might have experienced negative emotions, such as loss, aversion or anger. Although paired with different counterparts in different rounds, some trustors might have systematically decreased their expectations and investment in subsequent games (Tzieropoulos, 2013). Importantly, this effect is not as strong when the outcome is positive, suggesting an asymmetric impact of negative versus positive emotions on trust. While activating the rDLPFC can suppress negative emotions, inhibiting the rDLPFC plays the opposite role. This may suggest that under the negative emotion suppression mechanism, activating the rDLPFC can lead trustors to control the negative emotions related to loss, aversion or anger produced in the trust game. With fewer negative emotions, trustors are more willing to invest.

Our study parallels to two previous studies (Aimone et al., 2014; Zheng et al., 2017). Aimone et al. (2014) used fMRI and revealed that rDLPFC is pivotal in trust behavior. However, fMRI cannot demonstrate a causal relationship between rDLPFC and trust. Our study used tDCS and revealed the causal relationship. Zheng et al. (2017) showed that stimulation of rDLPFC cannot change interpersonal trust. In contrast, we conducted trust game experiment of multiround with role switching and recruited more subjects. The difference in experimental design allows the effect of rDLPFC to manifest in our experiments. Our study is a conducive supplement to their studies.

In trust games, the participants’ emotions may encompass betrayal aversion and guilt aversion. The impact of negative emotions (e.g., loss, aversion or anger) of betray on trust behavior is theoretically opposite to negative emotions (e.g., guilt). Numerous studies have explored participants’ feeling of guilt in trust game. Nihonsugi et al. (2015) found that anodal stimulation on rDLPFC significantly enhanced the guilt of trustee. However, similar to the study of Nihonsugi et al. (2015), most papers focused on the feeling of guilt of trustee in trust game (Chang et al., 2011; Engler et al., 2018; Shore and Parkinson, 2018; Cartwright, 2019). Our experimental design somewhat minimizes trustors’ feeling of guilt. First, our trust game consists of multiple rounds with different counterparts and without communication. When trustors opt not to transfer, they may not feel excessive guilt since they do not have to face the same counterpart in the subsequent rounds. Second, our experiment involves role switching. If trustors experience guilty for not to transfer in a prior round, they can mitigate this negative emotion by increasing the amount of transferring back as trustees. Thus, the guilt mechanism will not be the dominant driver in our experiment.

In addition to the fact that the DLPFC plays an important role in emotion control, the combined function of the DLPFC and the amygdala may also be the reason why enhancing DLPFC activity can increase trust. The DLPFC engages cognitive control. The amygdala is involved in processing social, emotional, and reward-related information. It is closely related to fear, anxiety and other negative emotions (Maren, 2001; Ipser et al., 2013; Berboth and Morawetz, 2021; Alexandra et al., 2022). Some studies have shown that downregulation of negative emotions, fear in particular, is involved in the combined action of the DLPFC and the amygdala. Specifically, cognitive reappraisal, a negative emotion regulation strategy, involves the PFC, which then modulates the amygdala (Berboth and Morawetz, 2021). There may be two ways that the DLPFC influences the amygdala. One is that the DLPFC engages the vmPFC, which then modulates the amygdala (Delgado et al., 2008; Hartley and Phelps, 2010), and the other is that the DLPFC modulates lateral temporal areas, which then indirectly influence the amygdala (Buhle et al., 2014; Kohn et al., 2014). In a study of behavioral experiments, Koscik and Tranel (2011) found that in a multiround trust game, participants with unilateral damage to the amygdala tended to show more trust in betrayals due to their benevolence, while neurologically normal adults tended to decrease trust in response to similar counterparts. In short, previous studies have suggested that negative emotions linked to the amygdala may be reduced via modulation by the DLPFC. In our trust game, when faced with negative results by trustees, trustors might have experienced a feeling of fear that they might face the same situation in later rounds and decided to reduce their subsequent investment. Thus, activating the rDLPFC functioned as a modulator of the amygdala and invoked cognitive reappraisal, leading to changes in negative emotional responses. Therefore, in our trust game, enhancing the activity of the rDLPFC increased trust compared to the sham group.

The results also showed that no significant difference was observed between the cathodal group and the sham group, although trust in the cathodal group was lower than that in the sham group. This outcome is common due to polarity-specific effects (Thair et al., 2017). Jacobson et al. (2012) carried out a meta-analytical review and found that anodal-excitation and cathodal-inhibition effects (AeCi) rarely occurred in cognitive research; that is, cathodal stimulation rarely works in inhibiting excitability. Filmer et al. (2014) found that cathodal stimulation does not significantly alter cognitive function. Polarity effects depend on various factors, such as age, sex, brain state and the preexisting regional excitability of each participant. Several studies applying tDCS over the rDLPFC also found no significant difference between the cathodal group and the sham group (Zheng et al., 2017, 2021b; Hu et al., 2022).

It can be drawn from the results of the five stages that activating or inhibiting rDLPFC activity in the first two stages could not alter participants’ trust, while activating the rDLPFC could significantly increase trust compared to inhibiting in the third and last stages, suggesting that trust is still a complicated kind of behavior. Another point was that participants’ mean trust in the cathodal group and the sham group experienced a sharp decline before the last stage and finally reached the lowest the last stage (McKenzie et al., 2016). Considering that investment may not result in the due return, participants were less willing to transfer their endowment.

## 5. Conclusion

In our study, we employed a multiround trust game and applied tDCS over the rDLPFC to determine a causal relationship between rDLPFC excitability and trust behavior. The results showed that on the whole, anodal stimulation increased participants’ trust compared with cathodal stimulation and sham stimulation. Thus, the rDLPFC plays an irreplaceable and significant role in trust behavior. However, the results in different stages were not completely the same, implying that the relationship between the rDLPFC and trust is still a complicated issue.

Although we found a causal relationship between rDLPFC excitability and trust, how the DLPFC affects trust remains unknown because trust behavior involves many factors, and the DLPFC has a wide range of functions. Future studies may improve the design of the experiment and focus on the mechanism by which the DLPFC affects trust. In summary, our work provides evidence of a causal relationship between rDLPFC excitability and trust behavior.

## Data availability statement

The raw data supporting the conclusions of this article will be made available by the authors, without undue reservation.

## Ethics statement

The studies involving human participants were reviewed and approved by the Zhejiang University of Finance and Economics Ethics Committee. The patients/participants provided their written informed consent to participate in this study.

## Author contributions

HZ and LS: designed experiment. HZ, LS, and LZ: performed experiment. LS and WZ: analyzed data. LS: drew figures. LS and HZ: wrote the manuscript. HZ, LS, WZ, JW, and XL: revised the manuscript. HZ and XL: finally approved the version to be published. All authors contributed to the article and approved the submitted version.

## Funding

This work was supported by National Natural Science Foundation of China [Grant Number: 71903169], Zhejiang Provincial Natural Science Foundation of China [Grant Number: LY19G030019], Zhejiang Province Philosophy and Social Sciences Key Research Base Project “Research on Sustainable Poverty Alleviation Boosting Mechanism” [Grant Number: 2022JDKTYB17] and Teaching Reform Program of the “14th Five-Year Plan” for General Undergraduate Colleges and Universities in Zhejiang Province “Visible + Realizable: An Exploration of Experimental Methods for Teaching Political Economy” [Grant Number: jg20220385].

## Conflict of interest

The authors declare that the research was conducted in the absence of any commercial or financial relationships that could be construed as a potential conflict of interest.

## Publisher’s note

All claims expressed in this article are solely those of the authors and do not necessarily represent those of their affiliated organizations, or those of the publisher, the editors and the reviewers. Any product that may be evaluated in this article, or claim that may be made by its manufacturer, is not guaranteed or endorsed by the publisher.
